# Warnock and its contested legacy in relation to donor conceived families: the case for regulatory reform

**DOI:** 10.1080/14647273.2025.2493252

**Published:** 2025-05-19

**Authors:** Caroline A. B. Redhead, Nicola Barker, Marie Fox, Lucy Frith

**Affiliations:** a Manchester Law School, Manchester Metropolitan University, Manchester, United Kingdom; b School of Law and Social Justice, University of Liverpool, Liverpool, United Kingdom; c Centre for Social Ethics and Policy, Department of Law, The University of Manchester, Manchester, United Kingdom

**Keywords:** Warnock Report, donor conception, direct to consumer genetic testing, law reform, donor anonymity, counselling

## Abstract

A generation on from the Warnock Report, the regulatory system it proposed remains largely intact, despite significant changes in the fertility sector, legal culture and wider society. In this article, we trace Warnock’s legacy, focusing on the context of gamete donor conception. Drawing on illustrative examples from the ConnectedDNA research project, we analyse two aspects of Warnock’s proposals - its recommendation that gamete donors should be anonymous and its key assumption that only the ‘triad’ of donor, recipient(s) and donor-conceived people have an interest in receiving information about each other. The jettisoning of donor anonymity coupled with a questioning of Warnock’s assumptions about the meaning of ‘family’, illustrate the challenges inherent in a key Warnock objective: to ‘future proof’ fertility law. Both the global market in gametes and embryos and the accessibility of Direct-to-Consumer Genetic Testing (DTCGT) technologies were wholly unforeseen by Warnock. Similarly, contemporary understandings of donation, families, kinship and relatedness exist in tension with Warnock’s original assumptions and, thus, with the principles underpinning the legislative framework. Given this, we recommend three specific reforms to the regulation of donor conception: (1) an urgent review and reformulation of information-sharing provisions, particularly with regard to donor-siblings; (2) an expansion of counselling and support provisions for those affected by donor conception; and (3) the effective imposition of a global ten-family limit. More generally, we suggest that piecemeal and *ad hoc* reforms to the Human Fertilisation and Embryology Act 1990 have often appeared contradictory and have failed to grapple with the global nature of fertility practice. Thus, we conclude by arguing that a comprehensive review of the legislative framework is needed to create a system of legal governance which meets the needs of the donor conceived community and remains fit for purpose in the twenty-first century.

## Introduction

Forty years ago, the Warnock Report (hereafter Warnock) laid the foundation for the regulation of assisted conception and embryo research in the UK (Department of Health & Social Security, 1984). Its recommendations were largely enshrined in the Human Fertilisation and Embryology Act 1990 (“the 1990 Act”) as originally drafted. Over a generation later, the key concepts and assumptions underpinning the UK’s approach to the regulation of medically assisted reproduction remain substantially intact, notwithstanding significant changes in the fertility sector, legal culture and broader society. As Emily Jackson has noted, this is largely attributable to the ‘flexibility [that] was ‘designed into’ the regulatory system proposed by Warnock’ (Jackson, 2022, p. 247). In this article, we examine Warnock’s legacy in the context of (gamete) donor conception, tracing its influence and evaluating its relevance in addressing contemporary challenges. We draw on examples from the ConnecteDNA research project to offer insight into how the current legal framework impacts those directly involved in donor conception. This UKRI ESRC-funded interdisciplinary project examined the use of online DNA testing by donor (egg, sperm and embryo) -conceived adults, donors and parents of donor-conceived people through sixty in-depth qualitative interviews, supplemented by a series of three workshops with stakeholder communities, including reproductive medicine clinicians, infertility counsellors and donor conception groups. These data informed the development of our argument. However, since this article is not intended as a report of the study, we draw only on select illustrative examples here (for a detailed account of the methods and study see Gilman et al., 2024).

In donor conception, with one significant exception pertaining to donor anonymity, Warnock’s key vision and its influence on the legal governance regime has endured for over three decades, notwithstanding the disruptive effect of advances in reproductive technologies and significant shifts in the wider legal context. In crafting its recommendations, Warnock was cognisant of the need to weigh future therapeutic or scientific advantages against present and future harm. It explicitly adopted a ‘steady and general point of view’ in attempting to ‘discover the public good’ on which the principles underpinning regulation of assisted reproduction would be based (Department of Health & Social Security, 1984, para 2). However, subsequent social and technological developments highlight the challenges inherent in its key objective to ‘future proof’ fertility law (see the Foreword to the Report: Department of Health & Social Security, 1984, para 1). We concentrate on two such developments in relation to donor conceived families. First, we analyse the legislative U-turn on one of Warnock’s key recommendations that, ‘as a matter of good practice any third-party donating gametes for infertility treatment should be unknown to the couple before, during and after the treatment’ (recommendation 18, Department of Health & Social Security, 1984, p. 82). Second, we focus on its assumption that, in the context of assisted conception, only the ‘triad’ of donor, recipient(s) and donor-conceived people have an interest in receiving information about each other. Warnock failed to consider the significance of wider genetic links, such as those between a donor-conceived person (DCP) and other DCPs born from the donation of the same donor (donor siblings), or between a DCP and the donor’s genetic relatives, including both a donor’s ‘own’ children and their wider family members.

Our contribution is to offer a socio-legal analysis which traces how, and with what implications, technological and societal change has moved beyond many of the principles and assumptions which informed the 1990 Act. We start by situating Warnock within its historical context, before reflecting on the evolving role of relationships and relationality in the landscape of fertility law and practice over time, and the growing emphasis on openness coupled with the significance of genetic links for many DCPs. We explore how contemporary understandings of donation, kinship and relatedness challenge the Committee’s original narrow assumptions about ‘family’ and, by extension, the framework of the 1990 Act. We then consider the impact of Direct-to-Consumer Genetic Testing (DTCGT) technologies, the advent of which could not have been predicted by Warnock. Similarly, the Committee could not have foreseen the impact of the online environment, which both facilitates the global trade in human gametes and information about human DNA and allows national laws and regulatory frameworks to be bypassed (Franklin & Lock, 2003; Vertommen et al., 2022). Tensions between the contemporary global reproductive trade and the 1990 Act’s historically situated thinking impact directly and indirectly on those affected by donor conception. Tension also exists between the parenthood provisions in the 1990 Act (as amended) and the Children Act 1989. Despite the 1990 Act’s stipulation that a donor will not be a legal parent, the Children Act allows for parental-like ‘rights’ to become available to donors who have established contact with a child conceived through their donation. Problematically, neither parents nor donors are usually informed of this possibility prior to conception, or prior to engaging with these technologies.

We conclude that such tensions, coupled with shifting societal attitudes, which themselves have been impacted by technological change, suggest the need for fundamental reform of the 1990 Act. However, this does not mean that all of Warnock’s recommendations and assumptions about donor conception are outdated. Some, notably the importance of counselling and family limits, are of *increasing* importance in the contemporary social, legal, and technological environment and should be strengthened. Further, given recent reports that sperm is exported once the 10-family limit is reached in the UK (Devlin, 2024a), we call for urgent clarification that this practice is currently prohibited by Regulations, pending more comprehensive reform.

## Warnock’s enduring but contested legacy

The Warnock Committee was convened to carry out a wide-ranging inquiry, guided by the following terms of reference:

To consider *recent and potential developments* in medicine and science related to human fertilisation and embryology; to consider what policies and safeguards should be applied, including consideration of the social, ethical and legal implications of these developments; and to make recommendations (Department of Health & Social Security, 1984 para 1.2, emphasis added).

In undertaking this work, the Committee was tasked not only with exploring how to regulate science and medicine at that time, but also with anticipating appropriate social, ethical and legal responses to discoveries and innovations yet to be made. In so doing, it aimed to frame recommendations that could be adapted to rapid scientific advancements and emerging ethical issues, noting that:

The pace of scientific discovery is unpredictable. Indeed, a number of major developments has taken place during the lifetime of the Inquiry. The changes which take place in society itself are also difficult to predict. The impact of scientific discoveries on the society of the future is therefore doubly hard to predict. *We took the pragmatic view that we could react only to what we knew, and what we could realistically foresee*. This meant that we must react to the ways in which people *now* see childlessness and the process of family formation, taking into account the range of views encompassed by our pluralistic society, the nature and value of clinical and scientific advances and the benefits of research (Department of Health & Social Security, 1984, p. 5, emphasis added).

The 1990 Act, which largely reflected Warnock’s proposals, was unquestionably a landmark piece of legislation. Underpinned by Warnock’s twin principles of ensuring consent to the use of donated gametes and embryos, and the welfare of children to be born via assisted conception (Department of Health & Social Security, 1984, paras 3.7 and 2.5 respectively), the 1990 Act established an expert regulator - the Human Fertilisation and Embryology Authority (HFEA) - to oversee any research or treatment involving creation of human embryos outside the body and the donation or storage of human gametes. In line with Warnock, this statutory regime is characterised by a broadly permissive approach, whereby only the most contentious aspects, such as human reproductive cloning, are prohibited; other types of treatment and research must be licensed by the HFEA. In 2008, Jackson noted that Warnock *‘*has had the most profound impact on the regulation of fertility treatment not only in the UK but worldwide’ and that the regulatory model it spawned ‘has proved to be sufficiently flexible and liberal to be able to accommodate scientific and social change, while also being sufficiently strict and rigorous to maintain public confidence’ (Jackson, 2008, p. 429). Yet, by 2022 she acknowledged that a statute based on recommendations made so long ago was ‘inevitably showing its age’ (Jackson, 2022, p. 233; see also Horsey & Jackson, 2023). Indeed, Brazier has suggested that constraints on the Committee meant, inevitably, that its recommendations would rapidly become outdated:

Warnock deliberated at a very early stage of the ‘reproductive revolution’. Neither the science nor the infrastructure which now underpins the ‘reproductive business’ was well developed (Brazier, 1999, p. 173).

Moreover, at the time the Committee was deliberating, legal understandings of family centred on (heterosexual) marriage. Children born outside of marriage experienced the stigma of ‘illegitimacy’, with associated legal disadvantages that were not abolished until the Family Reform Act 1987. Unmarried heterosexual couples were only beginning to receive limited legal recognition as ‘family’ (*Dyson Holdings v Fox* 1976) while the first recognition for same-sex families came considerably later (*Fitzpatrick v Sterling Housing Association* 2001). The enactment of ground-breaking legislation, including the Human Rights Act 1998, Gender Recognition Act 2004, Human Tissue Act 2004 and Civil Partnership Act 2004 was often accompanied by *ad hoc* amendments to the 1990 Act itself (Alghrani, 2018, p. 3). These post-Warnock legal developments reflected broader shifts in attitudes to assisted conception, families and secrecy (Blyth & Frith, 2015; Thompson, 2005). By 2004, the Government acknowledged that a review of the 1990 Act was necessary to respond to developments in reproductive medicine, changes in legal culture and the normalisation of assisted conception.

Yet, notwithstanding the scale of change, many contentious aspects of the 1990 Act were not considered in the first major review of the Act in the mid-2000s (Department of Health, 2005, para. 1.13; see also Department of Health, 2006). Hence, parameters were established to limit the debate and ensure that ‘the key question was not ‘what model of law do we want?’ but rather ‘what needs to be changed’’? (McCandless & Sheldon, 2010a). In consequence, the 2008 legislative reforms represented a missed opportunity (Alghrani, 2009; Fox, 2009). Reluctance to countenance radical reform similarly characterises the HFEA’s most recent consultation (Human Fertilisation and Embryology Authority, 2023a). The Authority stated that it, ‘did not seek to break with the broad settlement set out in the original Warnock Report’, but, rather, to update the settlement ‘for the needs of today’ and to ‘future proof’ the law as far as possible (Human Fertilisation and Embryology Authority, 2023b, Introduction). This continuing eschewal of radical, considered reform ignores the important changes to the post-Warnock legal landscape over time, which have had far-reaching effects for the donor-conceived community. While acknowledging that some of these technological innovations and regulatory challenges were beyond the Committee’s foresight (thereby illustrating the impossibility of ‘future-proofing’), we contend that there is now a compelling case to rethink completely the 1990 Act’s regime as it pertains to donor conception.

## Challenges to Warnock’s underlying assumptions

In relation to donor conception, the most significant departure from Warnock’s recommendations was the move to identity-release donation from April 2005. This followed growing calls to acknowledge DCPs’ rights to know their genetic origins for reasons of personal identity, health and well-being and ethical concerns about the potential psychological and emotional impact on DCPs unable to access this information (Frith, 2015). A judicial review in 2002 (*R (on the application of Rose) v Secretary of State for Health 2002*) confirmed that a right to obtain information about donors existed, and new regulations came into force in 2005 making it unlawful to use anonymously donated gametes in treatment in UK licensed clinics (Human Fertilisation and Embryology Authority (Disclosure of Donor Information) Regulations 2004). Consequently, those born from donations made after 1^st^ April 2005 can (with certain exceptions: see Human Fertilisation and Embryology Authority, 2023d) apply for *identifying* information about their donor when they turn 18 (non-identifying information is now available, on application, at the age of 16) (1990 Act, as amended, s31ZA). Importantly, no right exists to access information about other donor family connections, although upon adulthood, DCPs may access limited information (sex and year of birth) about donor siblings born from the same donor in a UK licensed clinic. Once they turn 18, DCPs may indicate to the HFEA that they wish to be contacted by donor siblings and join its voluntary contact register, the Donor Sibling Link (s31ZE). A donor has a limited right to know how many DCPs were born from their donation, the year of their birth and their sex (s31ZD). This represents a significant departure from Warnock’s recommendations and was seen by some commentators as evidencing societal recognition of a right or interest in knowing one’s genetic origins (Blyth & Frith, 2015; Wallbank, 2004). Importantly, this apparent concern with ‘truth’ about genetic origins does not yet extend to a legal obligation, which some scholars have advocated (Wade, 2020), to disclose to children that they were conceived using donated gametes. The HFEA’s Code of Practice (CoP) has, however, for many years advised clinicians to ‘encourage and prepare patients to be open with their children from an early age about how they were conceived’ (Human Fertilisation and Embryology Authority, 2023c, para 11.56).

The stories of participants in the ConnecteDNA research project indicate that, for many in the donor-conceived community (donors, DCPs and the parents of DCPs), genetic links to donor siblings and other donor family connections are significant, including, for some DCPs, during childhood. Thus, while other aspects of the traditional family have to some extent fallen away, genetic connection is arguably of growing importance.

### ‘Limited information-sharing’: the underlying concepts and assumptions

As noted above, one of Warnock’s key assumptions, linked to its support for donor anonymity and now increasingly challenged by evolving views on family and kinship, was that the circle of individuals entitled to receive information about one another in the context of assisted conception should be limited to the ‘triad’ of donor, recipient parent(s) and DCPs. We define this as ‘limited information sharing’. In unpacking this assumption, we address two related recommendations made by the Committee which, by contrast, are arguably more important now than they were when Warnock deliberated. These concern the need for a (global) limit on the number of DCPs born from one donor, and the crucial importance of counselling and support for *all* affected by donor conception, at *all* stages, including when information is released or contact requested.

In terms of its construction of family, Warnock was predominantly focused on the needs of a recipient heterosexual couple and the children born to them. This two-parent family with a father and a mother, sometimes described as the ‘sexual family’ (Fineman, 1995) was, ‘as a general rule’ considered to be better for children (Department of Health & Social Security, 1984, para. 2.11). Warnock’s narrow conception of the family has persisted, despite the removal of the requirement to consider the child’s need for a father in the 2008 reforms (McCandless & Sheldon, 2010a; Horsey & Jackson 2023). Warnock describes this family as a ‘valued institution’, ‘the place where … the child develops its own identity and feeling of self-value’ (Department of Health & Social Security, 1984, para 2.2). A gamete or embryo donor, or a surrogate, is characterised as a ‘third party’ who merely helps a couple to overcome their infertility (p.15, para 3.2). To protect the boundaries of the sexual family, and create *legal* distance between the donor and the DCP, Warnock recommended complete donor anonymity. This legal distance was perceived by Warnock to benefit donors (and, indirectly, the nascent fertility industry) by precluding the operation of any of the financial or societal obligations traditionally associated with fatherhood (for which donors would have been liable prior to the enactment of the Family Law Reform Act 1987, which was passed between Warnock reporting and the passage of the 1990 Act) (Department of Health & Social Security, 1984, para 4.9).

Warnock’s position aligned with the prevailing approach in adoption law at that time, which had only recently begun to transition away from the absolute secrecy enshrined in the Adoption Act 1949. Such secrecy had sought to prevent any involvement of birth parents, particularly single mothers, as ‘third parties’ who might disrupt the integrity of the nuclear adoptive family (Ryburn, 1995, pp. 161-2). While the Adoption Act 1976 permitted identifying information about birth parents to be released once the child reached 18, it was not until the Adoption and Children Act 2002 that the law began to encourage a more open approach (McFarlane, 2023), even making provision for post-adoption contact with birth families (Adoption and Children Act 2002, s26). Thus, at the time of Warnock there was a strong emphasis on maintaining the nuclear family unit free from external interference, which underpinned Warnock’s limited information-sharing framework.

Its recommendations were, therefore, made in the broader context of a transition from secrecy to (by the 1990s) limited openness about genetic origins in family law (Nordquist & Smart, 2014). While recognising that ‘it is wrong to deceive children about their origins’ (Department of Health & Social Security, 1984, para 4.12), Warnock believed that only very limited information about their donor’s ethnicity and genetic health should be given to the DCP on reaching adulthood (para 4.21). This limited information-sharing principle was enshrined in s.31 of the original 1990 Act. Clearly, then, persons to whom a DCP was genetically connected via their donor did not feature in Warnock’s understanding of family relationships. Further, Warnock assumed that DCPs would never be able to discover identifying information about their donor, so that no disruption of the DCP’s identity as a child of their legal parents would be possible. Today, the disruptive flexibility of the online environment created by DTCGT and social media platforms means that donor and donor-sibling anonymity can no longer be guaranteed (although, importantly, ConnecteDNA data show that DTCGT does not enable everyone successfully to identify their donor or any genetic relatives).

For the donor conceived community, the online sharing of genetic information has the potential to cause harm. We have written elsewhere about the experiences of DCPs who have discovered the circumstances of their conception following a DTCGT (Gilman et al., 2024; Redhead & Frith, 2024). Here, we focus on concerns about the additional harm of discovering large numbers of donor-siblings via DTCGT:

I don’t know if they actually took [donors’] specimens abroad but this clinic specifically, the doctor specifically and the branch specifically, they’ve been caught in the media resulting upwards of 250 children in the same city … so I’m worried about my sibling group being bigger than the recommended family limit, which is 10. I’m quite worried. [Anita, DCP] (participants have all been given pseudonyms).

The HFEA and other scholars have echoed this concern (Human Fertilisation and Embryology Authority, 2024a, see also Bauer & Meier-Credner, 2023).

It is this potential to generate anxiety and harm that underpins our suggestion that renewed attention should be paid to Warnock’s call for a limit on the number of families which can be formed from one donor’s gametes, and its recommendation that counselling and support be made available for the donor conceived community at all stages.

### Family limits and globalisation

Warnock suggested imposing a limit on the frequency with which donor gametes could be used, largely due to concerns about ‘the remote possibility of unwitting incest between children of the same donor, and … risks of transmission of inherited disease’ (Department of Health & Social Security, 1984, para 4.13). A limit of ten children was recommended, to be kept under regular review (para 4.26). The contemporary rationale for family limits, grounded in concerns expressed by the HFEA and the donor-conceived community about numbers of donor-conceived half-siblings and families that might be created (Devlin, 2024b; Human Fertilisation and Embryology Authority, 2024a), reflects another development which Warnock could not have anticipated. The globalisation of fertility practice through the growth in international sperm banks and related trade in donor gametes, often online, enables circumvention of jurisdiction-specific regulatory frameworks. This global market in gametes and increasing irrelevance of national boundaries in an online world was wholly unanticipated in the 1980s, and accordingly, Warnock did not specify whether the ten-child (later, ten family-) limit should apply globally or only within the UK.

The current HFEA CoP stipulates that UK clinics may not use donated gametes or embryos to create more than ten families (or any lower figure specified by the donor), although no limits exist on the number of children born within each family (Human Fertilisation and Embryology Authority, 2023c, para 11.56). Recent reports suggesting that UK clinics are now exporting sperm to international clinics once the ten-family limit is reached have generated concerns in the donor conceived community (Devlin, 2024a; see also a response to these concerns from the London Sperm Bank, 2024a). However, it is by no means clear that this practice is permitted by existing HFEA regulations. Schedule 2(1)(h) of the General Directions to clinics stipulates that:

the gametes or embryos *are not to be exported if they could not lawfully be used in licensed treatment services in the United Kingdom* in the manner or circumstances in which it is proposed that the gametes or embryos be used by the receiving centre (Human Fertilisation and Embryology Authority, 2021b, emphasis added).

Therefore, since it would not be lawful for clinics to use gametes or embryos to create a further family once the ten-family limit is reached in the United Kingdom, we take the view that it is unlawful for gametes or embryos to be exported for such use overseas. Consequently, UK clinics which export sperm once the 10-family limit is reached in the United Kingdom appear to be in breach of the Directions. This may be why, although UK clinics are explicitly required to tell prospective recipients of *imported* sperm that the ten-family limit only applies in the United Kingdom, no such explicit requirement exists for sperm that may subsequently be exported.

We suggest that a strong case exists for enshrining the ten-family limit in primary legislation rather than leaving it to Directions. Not only is enshrining policy in primary legislation symbolically important, but Directions/regulations have proven tricky for clinics and prospective clients to interpret. Still more importantly, primary legislation would override any potential or perceived conflict within the regulations. In the meantime, to avoid (further) confusion, we would strongly recommend that the HFEA CoP be revised to state explicitly that the ten-family limit should relate to the total number of families helped by the donation, globally.

It is likely that many recipient parents in jurisdictions such as the UK where a family limit applies were unaware that international sperm banks remain largely free to set their own limits on the number of families that can be created from one donor’s sperm and that, globally, large donor-sibling groups may exist. The European Sperm Bank (ESB) now states on its website that, on average, a donor helps 25 families worldwide. Noting that there is no international cap on the number of families a donor can help, the statement goes on to say that ESB has set a worldwide cap of 75 families for its donors (European Sperm Bank, 2024).

Back in 1984, although Warnock anticipated that there might be a case for a supranational approach to the regulation of fertility services, it concluded that such an approach ‘[would] be best formulated when individual countries are ready to pool knowledge and experience’ (para 1.8). In our view, in the context of family limits, a strong case now exists for imposing such supranational limits on the use of donor sperm. While we recognise that enforcement of cross-jurisdictional standards is challenging (Jansens et al., 2015), and that the HFEA has no authority over non-UK clinics, a supranational agreement would underline the requirement for UK clinics to include international conceptions within their ten-family limit and may help achieve consensus on desirable size of donor-sibling groups (see also the recent statement of the four Nordic National Ethics Councils, recommending a global limit: ETENE 2025).

### Counselling and support for DCPs

The second, related, Warnock proposal which we argue now warrants strengthening concerns counselling and support for donors, recipient families and DCPs. Warnock envisaged that non-directional counselling should be available to help donors, recipient parent(s) and ‘third parties at any stage of the treatment’ understand fully the implications of ‘what they [were] embarking on’, including where they might expect to experience difficulties (Department of Health & Social Security, 1984, paras 3.3-3.4). While the challenges experienced by those presently navigating the online environment in search of information about donor relatives were clearly not within the Committee’s contemplation, providing information and support remains essential today, albeit for different reasons (Wilde et al., 2014). For example, historic donors are often unaware that they might be identified via information accessed through ‘sleuthing’ within the online environment (Newton et al., 2023) Similarly, DCPs brought up in an environment of stigma and secrecy and kept unaware of the circumstances of their conception have no access to funded support to help them make sense of unexpected discoveries. Such uncovering of unknown genetic connections can result from use of DTCGT by the individual themselves or a a third party. Arguably, therefore, for donors, recipient parent(s) and DCPs, whether navigating these complex legal and online environments themselves or being impacted by the activities of others, access to counselling and support about the implications of donor conception has become more important than ever (Crawshaw et al., 2016). Yet, psychosocial support for those affected by donor conception continues to focus on the period surrounding treatment. Furthermore, the funded counselling and intermediary services which were available to donors and DCPs applying for information from the HFEA have, with effect from September 2024, been withdrawn, to be replaced by online advice and support (Crawshaw et al., 2024; Human Fertilisation and Embryology Authority, 2024b). This is regrettable, since the ConnecteDNA project shows that, for many participants, information available in the online environment, whether discovered intentionally or inadvertently, can cause significant psychological distress:

I didn’t want to believe it and […] then I thought what if my mum had an affair, so there was a lot of confusion and space and time kind of warped around that time, yes. And yes, it was very odd, very painful, and I started suffering from what I thought was shock, but now that I’m in therapy, I realise it was what they call complex PTSD… so I couldn’t complete my work and because you know, the layers were starting to hit, the waves of what happened, what really happened, I couldn’t perform to the best of my abilities…” [Anita, DCP]

Several interconnected issues are at play here. First, as discussed above, the 1990 legislative framework, underpinned by Warnock’s assumptions about what constitutes a ‘family’ (and, crucially, what does not) are out of step with current societal norms. The online environment enabling DTCGT (and other technologies) affords access to information outside the ‘official’ regulatory system. However, this often comes at an emotional cost, and, given the absence of social ‘scripts’ to manage the implications (Nordqvist, 2021), users lack recourse to appropriate support. The emotional and psychological considerations and the nature of support needed prior to and during contact between parents, DCPs, donors and donor family members differ significantly from the support currently offered by clinics prior to conception.

Consequently, we propose that all those affected by donor conception should have access to specialist support with appropriately qualified counsellors via the HFEA. It is vital, however, that the parameters of such support services are carefully defined, given empirical findings which show variations in practice across clinics with regard to pre-conception counselling (Lee et al., 2013; Sheldon et al., 2015).

Given the increasing use of DTCGT the implications of not providing appropriate support are significant. Our in-depth interviews exploring with donors, DCPs, and parents by donor conception their experiences of DTCGT have uncovered perceived tensions between the HFEA regulatory framework and the online and social worlds being negotiated. Careful consideration needs to be given to developing support and counselling which is appropriate to their needs as individuals navigate these processes, as well as to the most appropriate mechanism to fund and signpost such services.

## Donor conception experiences of (de-)anonymity through the online environment

Technologies available through the online environment, including DTCGT and social media data-sharing platforms, are outside of the HFEA’s regulatory remit and largely underpinned by commercial drivers, such as shareholder value. Subject only to light touch regulation, they enable users to circumvent the 1990 Act’s framework. DTCGT is a rapidly growing industry, estimated to be worth US$1.3 billion in 2023 and projected to reach US$3.4 billion by 2030 (Global Industry Analysts, 2024). Two platforms are widely used in the UK: Ancestry, which describes itself as the global leader in family history, and 23andMe. Ancestry claims to have more than 3.6 million subscribers and over 27 million people in its DNA network (Ancestry, 2025). 23andMe, which brands itself as a leading human genetics and biopharmaceutical company, offers DNA tests and health reports (although it is currently experiencing financial problems) (23andMe, 2024, 2025; D’Angiolo, 2025). Many providers purport to limit provision of DNA testing services to adult consumers (see, for example, Ancestry, 2024, paras 1.3 and 1.4) but, in practice, participants in the ConnecteDNA project found that they were able to make profiles for their infant children, and to share their child’s DNA on ‘matching’ databases.

While many ConnecteDNA participants expressed concerns about sharing their, or their child’s, DNA data with the service provider, they generally had no alternative route to access information about a donor or donor relatives at a time of their choosing. DNA data can be uploaded to a variety of databases, to increase the likelihood of ‘matches’ being found, and those searching for genetic relatives typically use DNA data in combination with extensive personal information publicly available on social media platforms and elsewhere to ‘triangulate’ people to whom they are, or might be, genetically related. DTCGT services interact with the regulation of fertility treatment when, via a DNA test (often marketed as a ‘fun’ product), a DCP discovers the circumstances of their conception. And it is through the ‘matching services’ offered by many providers of DTCGT (including Ancestry and 23andMe), often also mobilising information shared on social media, that DCPs can search for, find and sometimes contact their genetic relatives through donor conception (Gilman et al., 2024). As we have seen, this can be traumatic for DCPs and their families and result in donors being ‘outed’ where they have not disclosed their donation to their own families.

Thus, various discrete but overlapping legal frameworks have become relevant to the management of information relating to donor conception, often sitting in tension with each other as well as with the regulatory framework of the 1990 Act.

### The importance of donor relations

Our research findings show that DTCGT, combined with information on social media platforms, has changed how knowledge about donor conception is disseminated. While it is vital to recognise that participants’ experiences vary, DCPs who are (or become) aware of the circumstances of their conception as adults typically want information about their genetic relatives as soon as possible. Laura, a DCP, told us ‘I mean if someone had an envelope and said, “Here’s all the information,” oh, I would open that envelope in an instant’. Similarly, Mark, responding to a question regarding when he first started thinking of doing a DNA test said, ‘as soon as I found out [that I was donor-conceived], within a week I think I’d ordered a DNA kit’. These findings are reflected in other research which has explored the meanings attached to genetics by DCPs and the importance of ‘genetic thinking’ (Nordqvist, 2017) for a DCP’s identity (see also Indekeu & Hens, 2019 and Newton et al., 2023).

Many participants were interested in tracing their donor as part of understanding their own identity. Nick, whose donor had died before Nick found out that he had been donor conceived, stated:

‘I feel like I would have loved to have met him, and I feel like there’s a lot of similarities between, or some similarities anyway, between how he’s been described and how I, you know, was or am, and I feel like I’ve sort of missed out partly on that, and I do feel a bit like, you know, how would things have been different if I’d known who my biological father was and could relate to him’.

Yet, Nick’s donor’s family welcomed him into their world, and he was able to uncover other genetic connections through his donor, which became more significant to him than he had expected:

‘They sent me a lot of info [about the donor’s brother], my new biological uncle. They sent me photos, it was amazing to receive that, the similarity and you know, finally see I suppose a man in the family on my dad’s side who has those other features, and they told me about him, and there were lots of things that were similar … it felt somehow like, you know, that it was a genetic, hereditary similarity for me at the time, and then I really wanted to talk to this uncle’.

Some DCPs want to find donor siblings rather than their donor from the outset. For example, Vicky indicated that she was, ‘definitely more interested in finding out who my half siblings are’. Similarly, Bryonny, who was disappointed that no information was available through the official channels about the donor’s children, noted that they were ‘just as genetically connected as donor siblings … to all intents and purposes, half brothers or sisters genetically’. This feeling of connection (described by one participant as a ‘linky bond…’) was also experienced by another participant who was contacted by a DCP conceived from her father’s donation after her father died. Having initially questioned his story, she describes how ‘the evidence was building… and there’s this driving force that, I don’t know, it just kinda overtakes you, or did me. I just had to meet him really’.

Ruth wanted to find a donor-sibling for a different reason. She had always known she was donor conceived, ‘I was just always like drip-fed the information as soon as I could ask questions about where do babies come from’ and, ‘the question of what is family has always been like something discussed and something thought about’. However, as an adult she described a ‘moment of being cracked open’ followed by a ‘10 year-long journey, a lot of grieving, processing all sorts of different feelings’ around having had ‘without my own consent … half of my family disconnected from me’, wondering, ‘how did [my parents] not think that it would be important for me to know my biological family’? She remembers, ‘telling myself that “hey it would be cool just to find a sibling”‘ because ‘the prospect of actually finding my donor, I mean a biological father, it’s like too big to handle’. She felt that connecting with a donor sibling, ‘would be less overwhelming, in a sense, because the sibling wouldn’t know [the] biological father either’.

The significance of genetic connections can, however, also feel threatening, as in the case of one participant, Angela, whose husband had donated sperm before they met but had not told her. She explained:

‘I wasn’t expecting what happened, I was excluded from information. My children are going to be related to these people but I’m not. My husband is related to these people, but I’m not. They all look like each other, but I don’t. I’m completely excluded… that’s what I don’t like’.

In each of these examples, the importance of ‘linky’ genetic bonds, whether embraced or feared, is evident (see Nordqvist et al., unpublished). For some parents by donor conception, facilitating donor sibling relationships for their child during childhood takes on particular significance. Many ConnecteDNA participants described childhood as the key time for forming kinship connections and enduring bonds (see Gilman et al., 2025). For some, this was a reason to seek donor-sibling connections, while for others it underlined a strong objection to doing so in order to avoid ‘any of [the donor’s] family [finding] her and think[ing] they can have some kind of involvement’ (Ellie, parent by donor conception of an under five).

## ‘Linky bonds’ and (dis)connection: legal responsibilities towards donor-conceived children

The above quotes from DCPs allude to questions of the rights and best interests of donor-conceived children which, to date, have attracted little legal consideration (Adams et al., 2023). While Warnock was concerned that ‘due regard’ be had to ‘the interests of any child that may be born as a result [of assisted conception]’ (Department of Health & Social Security, 1984, para 2.7), it largely focused on the specific proposal that ‘as a general rule it is better for children to be born into a two-parent family’. Ultimately, this belief formed the basis for s.13(5) of the 1990 Act, which required account to be taken of the future child’s ‘need for a father’. This heavily criticised provision (Jackson, 2008) was amended (by s.14(2) Human Fertilisation and Embryology Act 2008 [‘the 2008 Act’]) to reference instead the need for ‘supportive parenting’. Yet, as McCandless and Sheldon (2010b) have argued, such reform did little to change how the welfare requirement was applied in practice (see also Sheldon et al., 2015). In continuing to protect donor anonymity during childhood and disregard the potential significance of donor-siblings, law once again fails to take account of the wider familial bonds and connections that we would argue can be in a DC child’s best interests. Data from the ConnecteDNA project highlight the tensions the current law generates between protecting children’s rights and promoting what parents perceive to be the best interests of DC children. This underlines earlier legal critiques of the welfare test and highlights the need for a fuller consideration of the best interests of DC children (Wade, 2020).

In the UK, DCPs now have the right to know their genetic origins once they reach adulthood but, currently, no right to access that information as children. The HFEA’s 2023 consultation asked respondents to consider whether the legislation should be amended to offer a ‘dual track’ system, which would give parents and donors a choice to opt for anonymity until age 18 or to permit identifiable information to be available on request after the birth of a child (Human Fertilisation and Embryology Authority, 2023a). In its subsequent proposals, however, the HFEA moved away from the dual track proposal. Noting that the availability of DTCGT and matching services ‘have revolutionised our ability to find genetic relatives’, it has recommended instead that legislative reform should remove donor anonymity from the birth of any DCP (Human Fertilisation and Embryology Authority, 2023b, Section 2). While it remains to be seen how such a change would be translated into practice, the HFEA clearly does not favour a ‘wholly’ open system of donor selection, where the identity of the donor is available before treatment. It states that further deliberation is needed on this point and on permitting a donor’s own children to access the Donor Sibling Link service. Moreover, while the issue of retrospective removal of anonymity has been mooted (Redhead & Frith, 2024), the HFEA appears to recommend ‘[c]ontinued respect of donor anonymity for pre-2005 donors and no retrospective early removal of anonymity for post-2005 donors’ (Human Fertilisation and Embryology Authority, 2023b, Proposal 8). This suggests that any legislative reform is likely to be approached cautiously.

### Evolutions in family law: from secrecy to (increased) openness

As discussed above, the case law relating to DCPs created through anonymous gamete donation focuses on the right of a DCP to know their genetic origins through identification of their donor (see *ex p Rose*, above) and has been grounded in an emerging right to identity (Wade, 2020; see also Brown & Wade, 2023). However, as also outlined above, the ConnecteDNA research found that, for some DCPs, connections between DCPs, donor siblings, and their donor’s wider relatives were at least as significant as identifying or contacting the donor (see discussion above; Gilman et al., 2024).

The current regulatory framework applicable to DCPs enables the creation of a genetic link but not, until adulthood, the possibility of a social and psychological relationship, albeit that in practice this is often subverted by the online environment. This contrasts with the current approach in the adoption context, where, although adoption functions to sever legal ties with birth families, the legislation provides for post-adoption contact (Adoption and Children Act 2002, s.26) in recognition of the importance of genetics to the adopted child’s identity. In relation to the online environment, Sir Andrew McFarlane (2023) has noted the potential dangers of a failure to share information about their genetic families with adopted children:

‘With the explosion of digital communication in the past two decades it is possible for an adopted child, quietly, alone in their bedroom, without the knowledge of their adopted parents, to trace and find their family. The temptation to do so, and then to make contact with them, must be almost irresistible. But the dangers of doing so, and the potential for significant emotional harm to result, are easy to contemplate.’

Donor conception differs in important respects from adoption, not least because most adoptions now take place due to concerns that the child is experiencing significant harm such that ‘nothing else will do’ besides their removal from their birth family (*Re B (A Child) (Care Order)* [2013], §198, per Lady Hale). Nevertheless, questions of identity and the importance of establishing one’s life story and making or preserving connections with those who share one’s genetics are of equal importance to DCPs, and the ConnecteDNA data confirms Sir Andrew’s concerns in the context of DTCGT. For example, ‘Charlotte’, a parent by donor conception, was conscious that communication with any ‘matches’ through genetic testing would need to be sensitively handled. She wanted contact, and to check that the matches were “nice people”, before telling her son about them.

In considering how best to protect DCPs from the harm of unintentionally learning their genetic origins via DTCGT, the comparison with adopted children may again be instructive. ‘Best practice’ in adoption requires that:

‘The issue of contact [with birth families] needs to be actively considered throughout the child’s minority…. Contact, where safe, appropriate and properly managed, can be valuable for an adoptive child, their new family and their birth family, including siblings and other relatives’ (Public Law Working Group, 2023; see also McFarlane, 2023).

We argue, therefore, that the framework of the 1990 Act (as amended) needs to adapt both to reflect changing ideas of family and kinship in contemporary society, including the importance of genetic (donor) links for some DCPs, and to minimise the need for people to turn to the under-regulated online environment in order to make connections. However, these connections must be chosen rather than imposed, and therefore some caution is needed as regards the potential risks to the donor conceived family of de-anonymising donors during childhood.

### A note of caution: the family law implications of contact with a donor during childhood

Notwithstanding a shift towards recognising non-genetic social and psychological parenthood (see for example, *Re G* (2006), §33-35, per Baroness Hale), family law continues to attach great significance to genetic/biological parenthood. This is particularly evident in cases involving known (i.e. non-anonymous) sperm donors. While the requirement to consider the child’s need for a father has been replaced, it appears that family courts still, in some cases, impose a father-like relationship for known donors.

In multiple cases involving lesbian mothers, known sperm donors who were not a legal parent of the child(ren) born from their donation – either because the donation was via a licensed clinic (s.28(6) 1990 Act) or because the birth mother was married (s.28(2) 1990 Act) – have been granted leave to apply for child arrangements orders (Children Act 1989, ss.8, 10(9)) when their relationship with the child(ren)’s legal parent(s) had become strained (see for example: *Re G; Re Z,* 2013; *Re X,* 2015). There are also examples of courts issuing child arrangements orders requiring donor conceived children to spend time with their donor (and even the donor’s parents) against the wishes of the child(ren)’s legal parents, even where the donor’s involvement had become ‘burdensome and troubling’ for the parents and they had sought to restrict contact (*Re G (A Child), 2018,* §9). In the recent case of *F v J, B and L* (2024), a man who had donated sperm to his friend via a licensed clinic was nevertheless able to acquire a child arrangements order, allowing him to regularly spend time with the child, and a specific issue order requiring the child’s mother to keep him informed of significant developments in the child’s life. Importantly, he also sought a parental responsibility order, which would have conveyed ‘all of the rights, duties, powers, responsibilities and authority which by law a parent of a child has in relation to the child…’ (Children Act 1989, s.3(1)). While this order was not granted, the sperm donor’s lack of parental status under the 2008 HFE Act was not a relevant factor in that refusal.

Such rulings have attracted deserved criticism for undermining the completeness of the lesbian-headed family (Yeatman, 2013). At a minimum, therefore, in the event that the law is reformed in line with the Human Fertilisation and Embryology Authority’s (2023b) proposal that donor anonymity be removed from the birth of a donor conceived child (discussed above), it seems imperative that parents (and, where applicable, children) be fully informed of the potential legal implications of a decision to establish contact with a donor during the DCP’s childhood. At present, the HFEA advises that donors via a licensed clinic will not ‘have any rights over how the child will be brought up’ (Human Fertilisation and Embryology Authority, 2021a), with clinics, in turn, giving similar advice to their clients. For example: ‘sperm donors do not have legal rights or responsibilities over any child born from their donation’ (London Sperm Bank, 2024b). However, as the cases above illustrate, this is not entirely accurate where a donor and child are known to each other when the child is under the age of majority. While the 1990 Act provides that a donor is not a legal parent (s.28(6)), the donor may still be able to exercise what would be colloquially understood as ‘rights or responsibilities over the child’. This is because, once a donor and child have established a relationship, should a dispute arise with the child’s legal parents, decisions about child arrangements and parental responsibility are based on the criteria set out in the Children Act 1989 and not on the parental status of the donor under the 1990 and 2008 Acts. The above cases suggest that the family courts see the maintenance of a genetic link to the donor and their wider family as being important to the welfare of the child, which is the paramount consideration in the Children Act.

There appears, in this line of case law, to be an underlying tension between the statutory aim of the 1990 Act (as amended) to create and recognise new family forms, particularly same-sex families (though note Horsey and Jackson’s (2023) argument regarding the very limited nature of the 2008 reforms), and a continuing cultural and judicial attachment to a genetic heteronormative understanding of family. Consequently, we argue that some caution is warranted when considering de-anonymising a donor’s identity during childhood, since the above cases suggest that the prospect of all donors becoming known, or knowable, could bring with it an imposition on donor-conceived families of relationships that they have not chosen. This suggests, perhaps, that Warnock’s intention to protect the boundaries of the sexual family through anonymity of the donor during childhood (or at least early childhood) retains some merit. There is, a difficult balance to be struck between recognising the importance of the DCP’s genetic links with the donor (and the donor’s family) and avoiding disruption to the integrity of the parent/child relationship by creating a father-like legal relationship between the donor and DC child. Arguably this issue has yet to be addressed by the family courts, so achieving such a balance requires specific consideration in any potential legislative reform.

While contact with a known donor can be positive and, as we have seen, for some DCPs is constitutive of their identity, we reiterate the importance for all parties, including the child, of access to implications counselling prior to contact, so that appropriate boundaries to the donor’s involvement can be discussed. Any potential reforms in this regard must also be informed by a full understanding of the potential family law implications for all parties should relationships between the donor and the parents break down during the DCP’s childhood.

## Conclusion

The legislative framework inspired by Warnock, which remains remarkably intact, has proven to be a ‘flexible and robust way to regulate a fast-moving area of science and clinical practice’ (Jackson, 2022, p. 232). Nevertheless, significant legal changes since 1990 have been *ad hoc* and sometimes contradictory (Miola, 2004, p. 67). Some have been prompted by individual litigation (e.g. concerning the right to know one’s genetic identity: Frith, 2015), others resulted from high profile campaigns on specific issues (Fox et al., 2009; Hervey, 1998) and still others reflect legal recognition of changing familial practices (McCandless & Sheldon, 2010a). Meanwhile the export of gametes in excess of national family limits and the use of DTCGT and online platforms to circumvent the 1990 Act’s limited information-sharing regime indicate that other practices simply elude legal controls. Consequently, the current unduly complex and unwieldy legislative framework needs reform, while more specific concerns regarding donor conception should be addressed. The significant inequities which now exist depending on whether DCPs were born with gametes donated pre-1990, between 1991-2005 or post April 2005 (Redhead & Frith, 2024) need to be remedied. Conversely, Warnock’s proposals pertaining to family limits and counselling *have* withstood the test of time and should now be strengthened and appropriately resourced.

Beyond these specific reforms, we concur with Brazier that ‘a statute enacted in 1990 no longer meets the needs of scientific developments’ and that its ‘paternalistic structure of regulation’ is ‘incompatible with a human rights culture’ (Brazier et al., 2023, 356). A re-evaluation of the current regulatory framework must also address the impact of the online environment and disruptive technologies, like DTCGT, on the DC community. There is a need for measures which either eliminate the need for reliance on technologies such as DTCGT or seek to bring them within an overarching legal framework that encompasses medical and digital technologies. Rethinking the governance of reproductive and genetic information in this way will require attentiveness to the implications for privacy, family dynamics and the best interests of DC children. Regulators will also need to grapple with the challenges of regulating beyond national boundaries and the implications of the family law jurisprudence involving known donors for the integrity of the parent/DCP relationship. The latter issue might, for example, require amendment to the Children Act 1989 to exclude donors from acquiring a parental responsibility order and give specific directions to the family courts that donors should not be granted a quasi-parental role through a child arrangements order.

As Warnock asked in 1984, and McCandless and Sheldon (2010a) later re-iterated, the key question remains what ‘model of law do we want’? In our view the legal framework should align with research which illuminates the lived experience of donor-conceived communities as this can inform more considered assessments of the best interests of donor-conceived children. To achieve this, a radical rethinking of the HFEA framework is now required.

## Acknowledgements

We would like to thank everyone who contributed to the research underpinning and the writing of this paper. First and foremost, we thank those who generously shared their time and experiences in interviews and stakeholder workshops for the ConnecteDNA study. We also thank the other members of the ConnecteDNA team (Leah Gilman, Nicky Hudson, Fiona MacCallum, Petra Nordquist, Jackson Kirkman-Brown and Anna Nelson) and Lucy Yeatman for helpful discussion and comments on the underpinning ideas. We are grateful to internal peer reviewers, Amel Alghrani and Danielle Griffiths, for insightful feedback and to the two anonymous peer reviewers and the editor for their input.

## Data Availability

The data that support the findings of this study are available on request from the corresponding author and will be archived.

## Case law

Re B (A Child) (Care Order) [2013] UKSC 33.

Re G (Children) [2006] UKHL 43.

Re G; Re Z (Children: Sperm Donors: Leave to Apply for Children Act Orders) [2013] EWHC 134 (Fam).

Re G (A Child) (Child Arrangements Order) [2018] EWCA Civ 305.

Re X [2015] 3 WLUK 872.

Dyson Holdings v Fox [1976] QB 503.

F v J, B and L [2024] EWHC 2802 (Fam).

Fitzpatrick v Sterling Housing Association, 2001[1] AC 27.

*Rose & Anor v Secretary of State for Health and Human Fertilisation and Smbryology Authority* [2002] EWHC 1593 (Admin).

## Legislation

Adoption Act 1949

Adoption Act 1976

Adoption and Children Act 2002

Children Act 1989

Civil Partnership Act 2004

Gender Recognition Act 2004

Human Fertilisation and Embryology Act 1990

Human Fertilisation and Embryology Act 2008

Human Fertilisation and Embryology Authority (Disclosure of Donor Information) Regulations 2004, SI 2004/1511.

Human Rights Act 1998

Human Tissue Act 2004
